# Comprehensive performance comparison of high-resolution array platforms for genome-wide Copy Number Variation (CNV) analysis in humans

**DOI:** 10.1186/s12864-017-3658-x

**Published:** 2017-04-24

**Authors:** Rajini R. Haraksingh, Alexej Abyzov, Alexander Eckehart Urban

**Affiliations:** 1 Department of Psychiatry and Behavioral Sciences, Stanford University School of Medicine, Stanford, CA 94304 USA; 2 Department of Genetics, Stanford University School of Medicine, Stanford, CA 94304 USA; 3 Department of Health Sciences Research, Center for Individualized Medicine, Mayo Clinic, Rochester, MN 55905 USA; 4 Program on Genetics of Brain Function, Stanford Center for Genomics and Personalized Medicine, Tasha and John Morgridge Faculty Scholar, Stanford Child Health Research Institute, 3165 Porter Drive, Room 2180, Palo Alto, CA 94304-1213 USA

**Keywords:** Copy Number Variation (CNV), Array Comparative Genome Hybridization (aCGH), SNP array

## Abstract

**Background:**

High-resolution microarray technology is routinely used in basic research and clinical practice to efficiently detect copy number variants (CNVs) across the entire human genome. A new generation of arrays combining high probe densities with optimized designs will comprise essential tools for genome analysis in the coming years. We systematically compared the genome-wide CNV detection power of all 17 available array designs from the Affymetrix, Agilent, and Illumina platforms by hybridizing the well-characterized genome of 1000 Genomes Project subject NA12878 to all arrays, and performing data analysis using both manufacturer-recommended and platform-independent software. We benchmarked the resulting CNV call sets from each array using a gold standard set of CNVs for this genome derived from 1000 Genomes Project whole genome sequencing data.

**Results:**

The arrays tested comprise both SNP and aCGH platforms with varying designs and contain between ~0.5 to ~4.6 million probes. Across the arrays CNV detection varied widely in number of CNV calls (4–489), CNV size range (~40 bp to ~8 Mbp), and percentage of non-validated CNVs (0–86%). We discovered strikingly strong effects of specific array design principles on performance. For example, some SNP array designs with the largest numbers of probes and extensive exonic coverage produced a considerable number of CNV calls that could not be validated, compared to designs with probe numbers that are sometimes an order of magnitude smaller. This effect was only partially ameliorated using different analysis software and optimizing data analysis parameters.

**Conclusions:**

High-resolution microarrays will continue to be used as reliable, cost- and time-efficient tools for CNV analysis. However, different applications tolerate different limitations in CNV detection. Our study quantified how these arrays differ in total number and size range of detected CNVs as well as sensitivity, and determined how each array balances these attributes. This analysis will inform appropriate array selection for future CNV studies, and allow better assessment of the CNV-analytical power of both published and ongoing array-based genomics studies. Furthermore, our findings emphasize the importance of concurrent use of multiple analysis algorithms and independent experimental validation in array-based CNV detection studies.

**Electronic supplementary material:**

The online version of this article (doi:10.1186/s12864-017-3658-x) contains supplementary material, which is available to authorized users.

## Background

Copy Number Variation (CNV) is a major category of human genetic variation. Most CNVs constitute normal variation and are functionally benign, while others have strong associations with disease (e.g. [1]). The accurate detection of CNVs is important for both biomedical research and clinical diagnostics. In this study, following our previous comparison of an earlier generation of arrays [2], we assessed the genome-wide CNV detection capabilities of all 17 commercially available high-density oligonucleotide arrays representing three different technologies: aCGH, (five Agilent arrays), SNP genotyping arrays (ten Illumina arrays), and combination aCGH/SNP platforms (two Affymetrix arrays). DNA from the extensively characterized genome of individual NA12878 (e.g. see 1000 Genomes Project, [3]) was hybridized to each array in two technical replicates. CNVs were then called twice for each replicate: first by using the respective manufacturer’s software for each platform, and second by using the Nexus Copy Number software by Biodiscovery for all platforms. Each call set was then compared to a gold standard set of CNVs for NA12878 that was derived from whole genome sequencing data produced by the 1000 Genomes Project. The gold standard contains only high confidence CNVs found by extensive sequencing of NA12878 and supported by multiple analytic principles (e.g. paired-end, split-read, or read-depth analysis), experimental validation (e.g. PCR or aCGH), or both [4].

Several technologies are now available for the unbiased and high-resolution detection of CNVs genome-wide. Generally, these technologies are either based on microarrays or ‘next-generation’ sequencing (NGS). Despite the expectation that eventually genome variation analysis will be accomplished using sequencing-based platforms only, the robust array-based methodologies are displaying a substantial amount of staying power. Arrays are currently fulfilling the near real-time needs of clinical cytogenetics and stem cell tissue culture laboratories [5, 6], as well as the requirement of typical genome-wide association studies to include thousands or even tens of thousands of samples in a cost-effective manner (e.g. [7–12]). In fact, microarray analysis has now replaced karyotype and FISH as the first-tier test for chromosomal aberrations in clinical cytogenetics [13]. At the same time, whole-genome sequencing-based analysis can easily overwhelm a typical laboratory’s workflow with very large amounts of raw data that require massive computational resources for data storage and processing as well as highly specialized bioinformatics skills for analysis.

The currently available array designs contain probe numbers that range from hundreds of thousands to several million. They also represent several design strategies such as a roughly equal genome-wide spacing of probes versus an evenly spaced backbone of probes in combination with higher probe density in exons or regions containing known CNVs. Here, we present an unbiased comparison of the CNV detection capabilities of all commercially available arrays including their sensitivities and size-resolutions.

## Results

### Gold standard CNVs for NA12878

**Fig. 1:**
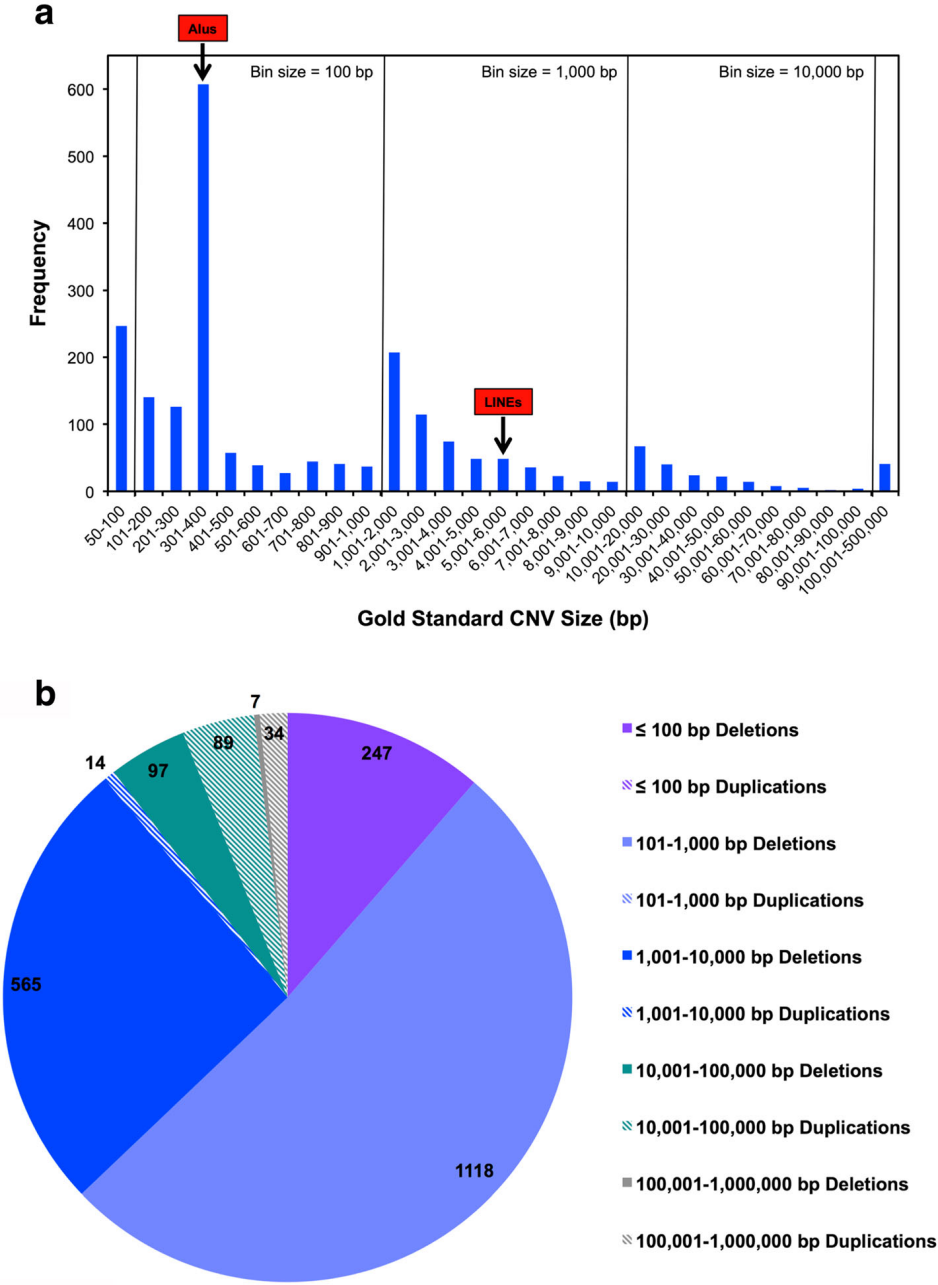
Size and nature of gold standard CNVs from sample NA12878. **a**. Histogram showing size distribution of NA12878 gold standard CNVs. Bin sizes change by a factor of ten across panels. Peaks in the size range of Alu elements, in the 301–400 bp bin, and in the size range of LINE1 elements, in the 5001–6000 bp bin, are indicated by arrows. **b**. Distribution of total numbers of gold standard deletions and duplications by size. It can reasonably be expected that most CNVs smaller than 1 kb in size are not detectable by the arrays in this study

Our gold standard set of CNVs for the genome of NA12878 was generated using the 1000 Genomes Project whole genome sequencing data [4]. It consists of 2171 CNV calls (2034 deletions and 137 duplications), 2076 of which are located on autosomes (1941 deletions and 135 duplications). The gold standard CNVs range from 50 bp to 453,312 bp, and 41 CNVs (7 deletions and 34 duplications) are larger than 100 kb [Fig. 1]. The bins with CNVs in the size ranges of Alu elements (301–400 bp) and LINEs (~6 kb) are labeled [Fig. 1a]. All gold standard CNVs ≤ 1 kb are deletions [Fig. 1b]. Most duplications (65%) are between 10–100 kb in size [Fig. 1b]. In total, 180 autosomal gold standard CNVs (54 deletions and 126 duplications) are polymorphic in the 1000 Genomes Project population and 33 of these (1 deletion and 32 duplications) are larger than 100 kb in size.

### Genome-wide CNV detection in NA12878 by 17 different microarray platforms

We analyzed the genome of NA12878 for CNVs using two technical replicate hybridizations for each of the seventeen different microarray platforms. Raw data from each experiment was collected from hybridizations performed either directly by the manufacturer or by manufacturer-recommended biotechnology service providers. For each platform, the raw data was analyzed separately using both the platform-specific manufacturer-provided software, at default settings for the main comparison, and the platform-agnostic Nexus software (Biodiscovery). The microarrays and their defining features, including total probe numbers, types of probes, probe spacing across the genome, source of the raw data, platform-specific analysis software, and total autosomal CNV calls using both analysis options, are summarized in Table 1. DNA from the well-characterized HapMap and 1000 Genomes Project individual NA10851 was used as a control in the Agilent aCGH hybridizations.

**Table 1:** Features and experimental details of CNV detection platforms used in this study

Array	Total Features	Probe Spacing	Data Source	Platform-Specific Analysis Software	Number of Autosomal CNVs Called (replicate 1, replicate 2)	
					Platform Specific Analysis	Nexus Analysis
Affymetrix Arrays						
Affymetrix CytoScan HD	2,600,000 Copy Number + 750,000 SNP	0.88 kb mean probe spacing in RefSeq genes	Affymetrix	Affymetrix Chromosome Analysis Suite	201,241	82,93
Affymetrix Genome-Wide Human SNP Array 6.0	946,000 Copy Number + 906,000 SNP	0.7 kb mean probe spacing	Affymetrix & Mc Carroll et al.	Birdsuite^a^	151^a^	105,112
Agilent Arrays						
Agilent SurePrint G3 Human CGH Microarray, 1×1M (Design ID 021529)	963,029 (60mers)	2.1 kb overall median probe spacing (1.8 kb in Refseq genes)	Service Provider	Agilent Genomics Workbench 7.0	124,133	65,53
Agilent SurePrint G3 Human High Resolution Microarray, 1×1M (Design ID 023642)	963,331 (60mers)	2.6 kb overall median probe spacing, 3 kb average probe spacing	Service Provider	Agilent Genomics Workbench 7.0	155,152	177,171
Agilent SurePrint G3 Human CGH Microarray, 2×400K (Design ID 021850)	411,056 (60mers)	5.3 kb overall median probe spacing (4.6 kb in Refseq genes)	Service Provider	Agilent Genomics Workbench 7.0	58,61	50,48
Agilent SurePrint G3 Human CNV Microarray, 2×400K (Design ID 021365)	442,892 (60mers)	1 kb overall median probe spatial resolution in CNVs	Service Provider	Agilent Genomics Workbench 7.0	365,365	433,489
Agilent SurePrint G3 Human CGH Microarray, 4×180K (Design ID 022060)	170,334 (60mers)	13 kb overall median probe spacing (11 kb in Refseq genes)	Service Provider	Agilent Genomics Workbench 7.0	24,25	30,32
Illumina Arrays						
Illumina HumanOmni5Exome v1	4,641,128	0.63 kb mean, 0.32 kb median probe spacing	Illumina	Illumina GenomeStudio 2011.1 and cnvPartition 3.2.0	426,397	66,62
Illumina HumanOmni5-4v1	4,301,331	0.68 kb mean, 0.36 kb median probe spacing	Illumina	Illumina GenomeStudio 2011.1 and cnvPartition 3.2.0	63,61	56,61
Illumina HumanOmni25Exome-8v1	2,583,651	1.13 kb mean, 0.57 kb median probe spacing	Illumina	Illumina GenomeStudio 2011.1 and cnvPartition 3.2.0	58,57	33,35
Illumina HumanOmni25-8v1-1	2,338,671	1.25 kb mean, 0.66 kb median probe spacing	Illumina	Illumina GenomeStudio 2011.1 and cnvPartition 3.2.0	31,34	28,24
Illumina HumanOmni1Quad-v1^b^	1,134,514	1.2 kb mean probe spacing	Service Provider	Illumina GenomeStudio 2011.1 and cnvPartition 3.2.0	245,263	139,143
Illumina HumanOmniExpressExome 1.2	964,193	3.0 kb mean, 1.4 kb median probe spacing	Illumina	Illumina GenomeStudio 2011.1 and cnvPartition 3.2.0	31,34	17,17
Illumina HumanOmniExpress-24v1-0	730,525	4.1 kb mean, 2.2 kb median probe spacing	Illumina	Illumina GenomeStudio 2011.1 and cnvPartition 3.2.0	15,12	11,10
Illumina HumanCoreExome v1.1	547,644	5.3 kb mean, 1.9 kb median probe spacing	Illumina	Illumina GenomeStudio 2011.1 and cnvPartition 3.2.0	5,4	7,6
Illumina CytoSNP-850 K	850,000 (50mers)	50 kb mean probe spacing, 10–20 kb in 3,262 cytogenetically relevant genes	Illumina	Illumina GenomeStudio 2011.1 and cnvPartition 3.2.0	12,17	25,21
Illumina PsychArray	571,054	5.5 kb mean probe spacing	Illumina	Illumina GenomeStudio 2011.1 and cnvPartition 3.2.0	5,4	10,9

^a^ The platform specific software CNV call set for the first replicate of the SNP 6.0 array was obtained from published data [19]. The second replicate of the SNP 6.0 array was only analyzed using Nexus software as per the Affymetrix recommendation for analysis of this array

^b^The Illumina HumanOmni1Quad array has been recently discontinued but was included in this study as this array was widely used previously and many investigators may be reanalyzing data from this array for copy number information

We also hybridized the genome of NA12878 on the newest Illumina array, the Infinium Multi-Ethnic Global-8 v1.0 array (commercial version of MEGA EX array) in replicate and called CNVs using the Nexus and Illumina CNVPartition algorithms with default settings. No CNVs were called using either algorithm in this sample and so this array was excluded from our comparison. These results are discussed in the Supplement.

### Comparison of array CNV calls to gold standard CNVs

**Fig. 2:**
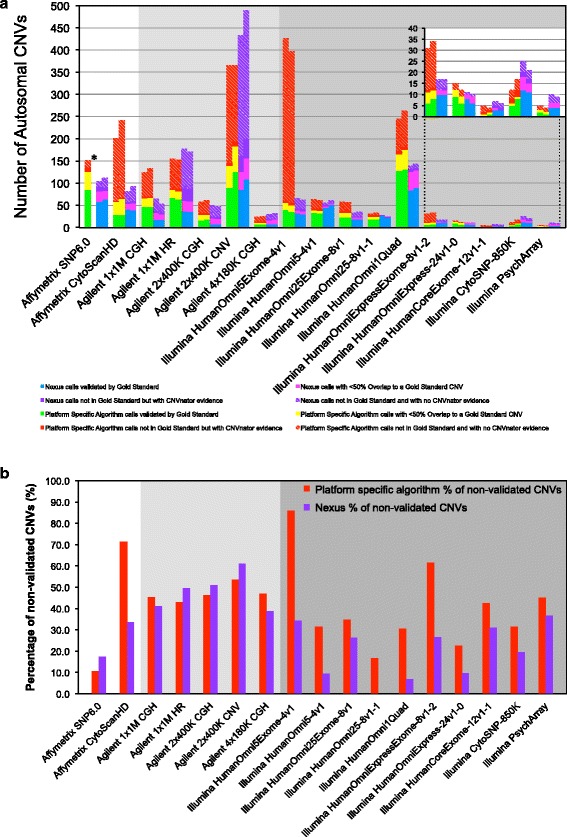
CNV detection performance of each array using two different algorithms. **a**. Overlap of autosomal CNV calls from two different algorithms for each array platform with gold standard CNVs. Data is shown for each of two technical replicates per array. CNV call sets derived from the platform specific algorithm are in *green*, *yellow*, and *red*, and those derived from Nexus are in *blue*, *pink*, and *purple*. The number of array CNV calls overlapping a gold standard CNV by 50% reciprocally in size is in *green* and *blue*, by less than 50% reciprocally in size is in *yellow* and *pink*, and not overlapping a gold standard CNV is in *red* and *purple*. Array calls not overlapping a gold standard CNV at all were further analyzed for sequencing-based confirmation using CNVnator generated CNV calls based on the 1000 Genomes Project sequencing data for NA12878. The number of CNV calls not overlapping a gold standard CNV but with CNVnator support is shown as *solid red* or *purple* bars. The number of CNV calls not overlapping a gold standard CNV and with no CNVnator support is shown as hashed *red* or *purple* bars. **b**. Average rate of non-validated CNV calls for each array platform and for each algorithm. The rate of non-validated calls is calculated as the percentage of the total number of CNVs called from an array that do not overlap a gold standard CNV and do no have any supporting evidence from CNVnator (hashed *red* and *purple* bars in a.). Average rate of non-validated calls is based on two technical replicates

The genome-wide CNV calling abilities of each array were assessed by determining the number of NA12878 gold standard CNVs it was able to detect [Fig. 2]. A CNV called by an array is considered valid if either it overlaps a single gold standard CNV by ≥ 50% reciprocally in size, or there exists a set of gold standard CNVs such that each event has ≥ 50% overlap with the platform CNV call, and ≥ 50% of the platform CNV overlaps with this set of CNVs. For all arrays, there were considerable numbers of CNV calls that did not meet these criteria.

In order to assess the validity of these remaining CNV calls, we used two less stringent analytical criteria. We calculated the number of CNVs that overlapped the gold standard CNV < 50% reciprocally. This rescued some of the platform CNV calls that did not meet the 50% reciprocal overlapping criteria, but not all. Our gold standard set of CNVs is robust but incomplete i.e. there may be additional true CNVs in the NA12878 genome, many of which may be detectable by some of the arrays, which did not meet the stringent requirements to be included in the gold standard. Therefore, we tested whether 1000 Genomes Project deep sequencing data alone supported the validity of array CNV calls that do not overlap gold standard CNVs at all. We employed read-depth analysis using the CNVnator algorithm [14] to generate CNV calls using 1000 Genome Project deep sequencing data (2×250 bp paired-end sequencing to 60× coverage on Illumina HiSeq) for NA12878 and counted how many of the remaining platform CNV calls overlapped a CNVnator call using the 50% reciprocal overlapping criteria described above [Fig. 2a, Additional file 1: Table S1].

The various arrays detected vastly different total numbers of autosomal CNVs, ranging from 4 (Illumina HumanCoreExome and Psych arrays using Illumina analysis software) to 489 (Agilent 2×400K-CNV microarray using Nexus analysis software) [Table 1 and Fig. 2a]. Consequently, the total number of validated autosomal CNVs for each array also varied widely. In general, among arrays from the same manufacturer the total number of called and validated autosomal CNVs increased with increasing probe number but there were some notable exceptions. Among the Agilent arrays, designs targeting known genes or CNV regions in addition to a substantial genome-wide ‘backbone’ (1×1M-HR and 2×400K-CNV respectively) detected many more CNVs than arrays with the same or an even larger number of probes but with an even probe spacing (1×1M-CGH and 2×400K-CGH) [Fig. 2a]. The widely used but now discontinued Illumina HumanOmni1Quad array contains ~1 million probes including dense CNV-specific probes in common CNV regions. This array called significantly more total and validated CNVs than most other HumanOmni arrays containing either ~2.5 million or > 4.7 million probes without CNV-specific probes. The only HumanOmni array design that called more CNVs than the legacy Omni1Quad, the HumanOmni5Exome, when analyzed by the manufacturer-provided software, produced a very large number of CNV calls that were not validated using our criteria. Importantly, the CNV detection performance of each array was consistent between replicates regardless of the algorithm used [Fig. 2a]. Technical replicates using the same algorithm produced very similar total numbers of CNVs (standard deviation < 9 for most arrays except Agilent 2×400K-CNV with a standard deviation = 26 (platform specific) and 40 (Nexus)) as well as very similar numbers of validated CNVs within each validation category.

### Algorithm choice and parameter settings can dramatically affect CNV results

In some cases, the same raw data analyzed with different algorithms resulted in considerably different numbers of total and validated CNV calls [Table 1 and Fig. 2a]. For all arrays except the Agilent 2×400K-CNV array, Nexus produced fewer total but as many validated calls as the platform specific algorithms. Therefore, Nexus analysis generally resulted in lower rates of non-validated calls for the arrays in this study [Fig. 2b]. The most striking difference between employing the Nexus and platform specific software is observed with the Illumina Omni5Exome array. For this array Nexus called an equivalent number of autosomal CNVs as it did for the similarly sized Omni5Quad array, but the platform specific algorithm called almost eight times more. Consequently, we computed rates of non-validated calls of 86% (platform specific algorithm) versus 31% (Nexus) for the Omni5Exome array [Figs. 2a and b].

Using a particular algorithm but with different parameter settings on the same raw data also produced notably varied results [Additional file 2: Figure S1]. Reducing the stringency of the parameters resulted in appreciably more total but only marginally more validated CNVs calls. These two phenomena have been well documented for previous generations of arrays and our results for the currently available arrays are consistent with the published literature.

### Size distributions of validated and non-validated array CNVs

In general, the arrays with more probes and the CNV focused designs (Affymetrix SNP6.0, Agilent 1×1M-HR, 1×1M-CGH, and 2×400K-CNV, and Illumina HumanOmni5Exome and Omni5) showed no difference in the size distributions of the validated and non-validated CNV calls indicating that size is not the distinguishing factor here. However, for arrays with less probes and even probe spacing across the genome (Affymetrix CytoScanHD, Agilent 2×400K-CGH, Illumina HumanOmni2.5Exome and smaller) the validated CNV calls are significantly larger than the non-validated CNV calls [Additional file 3: Figure S2].

### Exome content on arrays increases number of non-validated CNVs calls

The Illumina HumanOmni5, HumanOmni2.5, and HumanOmniExpress each form the basis of a corresponding exome-enriched design (HumanOmni5Exome, HumanOmni2.5Exome and HumanOmniExpressExome respectively) that contains an additional ~500,000 exome-specific probes. We observed considerably higher rates of autosomal non-validated calls for exome-enriched designs compared to their non-exome-enriched versions, which achieved some of the lowest rates of non-validated CNVs in this study [Fig. 2b]. Non-validated rates of 86% for the HumanOmni5Exome versus 31% for the HumanOmni5, 35% for the HumanOmni2.5Exome versus 17% for the HumanOmni2.5, and 62% for the HumanOmniExpressExome versus 23% for the HumanOmniExpress array were computed when using the platform specific algorithm. Absolute rates of non-validated calls were much lower when using the Nexus algorithm but followed a similar trend. However, the total CNV call rate was also lower when using the Nexus algorithm. Conversely, for the Agilent 1×1M designs, the 1×1M-HR array, which specifically targets known genes, called substantially more autosomal CNVs (25 using the platform comparison software and using Nexus) than the 1×1M-CGH design yet the rates of non-validated calls for both arrays were similar.

### Cytogenetic array designs balance comprehensive CNV detection and low rates of non-validated calls to different extents

Among the arrays specifically designed or commonly used for cytogenetic purposes (Affymetrix CytoScan HD, Agilent 4×180K-CGH, and Illumina CytoSNP-850), the CytoScan HD produced the highest number of validated CNV calls (57–67 valid calls) compared to the Agilent 4×180K CGH (13–20 valid calls) and Illumina CytoSNP-850 (8–20 valid calls). However, the approximately three times as many validated calls from the Affymetrix array were accompanied by a high rate of non-validated calls. Using the respective platform specific algorithms, the Affymetrix array produced a rate of non-validated calls of 71% compared to 47% for the Agilent array, and 31% for the Illumina CytoSNP-850 array. Using the Nexus algorithm, the Illumina array still produced the lowest rate of non-validated calls of 20% versus 34% and 39% for the Affymetrix and Agilent arrays, respectively, though differences in the rates were less pronounced.

89% (platform specific algorithm) and 93% (Nexus) of non-validated CNV calls from the Affymetrix CytoScanHD array were ≤ 25 kb in size. Our results are consistent with the advertised size range of reliable CNV detection of 25–50 kb. 100% of the non-validated calls obtained by the Illumina CytoSNP-850 array, using both the platform specific and Nexus algorithms, were ≤ 25 kb in size [Additional file 3: Figure S2]. This array was designed to maximize calls within regions that are associated with genetic disorders, including 3,262 genes of known cytogenetic relevance, whilst minimizing false calls, noise in the data, and the detection of common polymorphisms. It has an average resolution of 10–20 kb in the targeted regions and 50 kb in the backbone. This design strategy likely explains the small number of calls in the presumed healthy genome of NA12878 and the relatively low rate of non-validated calls. Our data suggests that these two arrays can be used to reliably detect CNVs ≥ 25 kb, and that calls ≤ 25 kb in size (and not in the targeted regions for the Illumina array) should be either validated by orthogonal experimental means or disregarded. 90% (Platform Specific Algorithm) and 75% (Nexus) of non-validated calls obtained from the Agilent 4×180K array were ≥ 25 kb in size and as large as ~200 kb (Platform Specific Algorithm) and ~1 Mb (Nexus). These findings reflect the reduced resolution of this array based on its much smaller probe number and median probe spacing of 13 kb.

### Most arrays detect only one of seven gold standard deletions larger than 100 kb

**Fig. 3:**
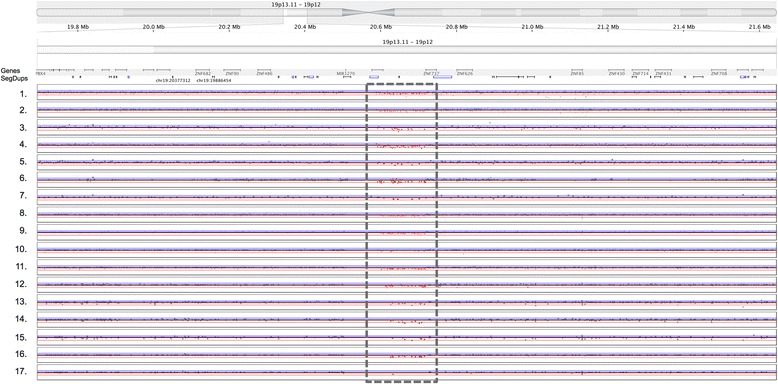
Detection of a 122 kb gold standard deletion on chromosome 19p by 17 arrays. *Horizontal axis* shows position along chromosome 19. *Vertical axes* show log R ratio of fluorescence of NA12878 DNA over fluorescence of reference DNA. *Grey dots* indicate probes that have not been called as part of a CNV. *Red dots* indicate probes that have been called as part of a CNV. *Horizontal lines* indicate Nexus cutoffs for low and high copy deletions (*red*) and duplications (*blue*). *Gray* dashed box indicates CNV region. Genes and segmental duplications (SegDups) are also shown. 1. Affymetrix SNP6.0, 2. Affymetrix CytoScanHD, 3. Agilent 1×1M-CGH, 4. Agilent 1×1M-HR, 5. Agilent 2×400K-CGH, 6. Agilent 2×400K-CNV, 7. Agilent 4×180K-CGH, 8. Illumina HumanOmni5Exome, 9. Illumina HumanOmni5, 10. Illumina HumanOmni2.5Exome, 11. Illumina HumanOmni2.5, 12. Illumina HumanOmni1Quad, 13. Illumina HumanOmniExpressExome, 14. Illumina HumanOmniExpress, 15. Illumina CoreExome, 16. Illumina CytoSNP-850, 17. Illumina Psych Array

All arrays analyzed here, based on their total probe counts and average probe spacings, are expected to call the seven gold standard deletions that are ≥ 100 kb in size. However, only one such deletion was detected by most arrays using Nexus [Fig. 3]. This 122 kb deletion on chromosome 19p2 partially overlaps two genes, *ZNF826P* and *ZNF737*, and one microRNA, *MIR-1270*. Four deletions ≥ 100 kb, two on chromosome 4 and one each on chromosomes 6 and 8, were not found by any aCGH platforms but were found by the other platforms. This is because the genomes of both NA12878 and NA10851 (i.e. the aCGH control DNA sample) have the same copy number at these loci per the 1000 Genomes Project. One deletion on chr3:162514471–162625647 was only called by the aCGH technologies and not by any other platforms due to a lack of probes in this region. There are several SNPs with > 1% allele frequency [dbSNP – UCSC genome browser] that could have formed the basis for probes. But this region may have been excluded from the design because there are no known genes and the sequence appears quite repetitive. In other cases the deletions were found by the arrays with more probes while arrays with less probes lacked enough coverage in these regions to make robust calls. Additional file 4: Table S2 summarizes the detection of these seven large deletions by each array using Nexus.

## Discussion

To our knowledge, this study represents the only comprehensive assessment of all currently available high-density oligonucleotide microarrays capable of genome-wide CNV detection. By analyzing the abilities of each array to detect a gold standard set of CNVs in the well-characterized genome of NA12878, we showed that the current generation of array designs encompasses powerful tools for CNV analysis in the human genome but still required a careful quantitative comparative analysis for researchers and clinicians to be able to select the appropriate tool for a given application. Furthermore, numerous studies originally designed for SNP analysis only (i.e. GWAS type studies) have been carried out on some of these arrays or their predecessors, and these data could be further analyzed for CNVs. The present work helps to contextualize the actual or potential CNV findings of hundreds of publicly available datasets.

Our study confirms and quantifies some general expectations and also reveals some unexpected findings that could prove valuable in designing array-based CNV-discovery studies. In general, more powerful arrays can be designed using more probes. But array design strategy proved to be a potentially at least as important feature than probe number for CNV detection. Deviating from the simplest design strategy of even probe spacing along the genome can yield both beneficial and detrimental consequences. Increasing probe densities in known CNV regions of the genome, in combination with a sufficient genome-wide backbone of probes, generally leads to more detection power. However, if the backbone coverage is not sufficient or regions such as gene deserts are devoid of probes, the design may not detect even some relatively large CNVs. Large CNVs in gene deserts may still be biologically relevant, for example they may be potentially associated with molecular and phenotypic effects that could be transmitted by changes in chromatin conformation. Therefore, it would be suboptimal to design a discovery study using tools that would make it difficult to detect such CNVs.

Unexpectedly, we found that the additional exome content on Illumina Omni arrays had no clear benefit for CNV discovery. We observed a dramatic increase in non-validated CNV calls without a corresponding gain in validated calls compared to the same array designs without the exome content. Though the unwanted effects of the exome-probes could be somewhat ameliorated by computational means, the use of arrays with this specific exome content for CNV discovery should be cautiously considered. Also, this observation illustrates why the use of two or more independent analysis algorithms and combining their results is strongly recommended. However, we should not draw the general conclusion that all gene-specific content leads to high rates of unvalidated calls. For example, the gene-targeting design of the Agilent 1x1M-HR design compared to that of the 1×1M-CGH design with more evenly spaced probes led to increased CNV detection with no change to the rate of non-validated calls.

The use of various algorithms, software packages, and parameter settings for the analysis of the same data is a topic of considerable importance for array-based CNV detection. For example, previous work has shown that there is more variability in CNV calls from different algorithms using the same raw data than from the same algorithm using raw data from different labs [15]. Our results are consistent with this finding since we showed cases with strikingly different results from using different algorithms on the same raw data. Nevertheless, the overall performance trends we observed across the various arrays were independent of the analysis algorithm and this provides more validation for our approach. Furthermore, our data confirmed the previously observed phenomenon that, just as using different algorithms, parameter-tuning using a single algorithm can also produce dramatically different CNV results based on the same raw data. A comprehensive comparison of the numerous software packages available for CNV analysis of the arrays discussed here was beyond the scope of our study. Thus, we would recommend a similar comparative analysis if researchers are considering using an algorithm that was not analyzed here.

The arrays in this study can be classified as either cyto-designs for the confident detection of large aberrations, or high-powered arrays including high-resolution aCGH and high-density SNP arrays, that offer more comprehensive discovery of CNV across a wider range of sizes but with the caveat that additional caution is to be applied in the interpretation of the findings. Cyto-designs are generally sufficient for cytogenetics studies or clinical practice but are likely to miss medium-sized or smaller but still potentially functionally relevant CNVs. Thus for association or discovery studies, high-powered arrays should be used and augmented with substantial orthogonal experimental validation when using arrays with high false discovery rates especially in small size ranges.

Our gold standard, derived from whole genome sequencing data, is a high-confidence set of CNVs for NA12878 based on the state of the art. Additional high-confidence CNVs, particularly duplications, will continue to be defined for this genome. For example there is currently an effort by the National Institute of Standards and Technology to create a standard map of copy number variation by integrating CNVs found in the genome of NA12878 based on numerous data from the 1000 Genomes Project, the Illumina Platinum Genomes, Complete Genomics, BioNano, and microarrays. However, we expect that additions to the gold standard will not fundamentally alter the results of this study regarding the power of the various array designs for detecting CNVs.

Given the many factors and scenarios that researchers will be facing when deciding which array to choose, it is not appropriate for us to determine a ‘winning’ platform or array design. In some scenarios the data may have been created already while in others the essential instrumentation for a given platform (e.g. platform-specific robotics or scanner) may already be present at an institution. Then there will be studies with relatively small sample size where choosing a more densely tiled and more expensive array design will be feasible, while for other studies it will be advantageous to choose a more affordable array to be able to analyze a larger cohort. There are many combinations of these factors and scenarios, and we expect that our present analysis will contribute to successful study designs. In this context it is important to emphasize the following additional factors for such studies. Whenever possible it is advisable to analyze array data with more than one algorithm in parallel and to give higher weight to CNVs that are located in the overlap of results. Experimental validation of CNV calls with orthogonal methods such as qPCR, ddPCR, FISH or, if possible, whole-genome sequencing of a subset of samples is of very high importance. Such validation is essential before reporting novel CNVs. And, it should still be strongly considered when the findings consist of previously reported CNVs called anew by only a single algorithm, or are of a medium or small size.

## Conclusions

Microarray-based analysis of copy number variation is a powerful tool for genome analysis. The current generation of available arrays has further enhanced this power and ensured that this technology will remain useful for years to come. However, the successful deployment of this technology for a given application is critically dependent on the selection of an appropriate platform and array design. Our results indicate that to maximize the validity of CNV detection by these arrays, the use of more than one CNV calling algorithm during data analysis and appropriate extensive experimental validation with orthogonal techniques should be considered. Our study represents a widely applicable resource highlighting the benefits and limitations of the various arrays for others who are utilizing these for CNV analysis in the context of large-scale association studies, genome-characterization, or clinical cytogenetics applications.

## Methods

### Sample selection

The genomes analyzed in this study were selected from the 1000 Genomes Project [3] and previously from the International HapMap Project [16]. The test sample, NA12878, was selected due to extensive prior characterization of its genomic variation by the 1000 Genomes Project, ultra-high resolution array Comparative Genome Hybridization [17], and its use as a standard for the study of human genome variation by the National Institute of Standards and Technology’s Genome in a Bottle project [18]. NA12878 is a Utah resident of European Ancestry (CEU) and is the daughter in one of the two trios sequenced at high coverage in the 1000 Genomes Pilot Project. The control sample NA10851 was also chosen because of extensive genomic characterization including its use as the control in ultra-high resolution aCGH and as a 1000 Genomes Project low coverage sample [3, 17]. NA10851 is a male of European Ancestry from Utah (CEU). Genomic DNA for these samples as well as appropriate approval for this study was obtained from the Coriell Institute for Medical Research.

### NA12878 Gold Standard CNVs

The gold standard CNVs utilized in this study were a subset of those CNVs released by the 1000 Genomes Project from 8-fold coverage Illumina paired-end population-scale sequencing data (available at 1000genomes.org) and analysis of the genomes of 2,504 individuals [4]. CNV discovery was conducted using the following tools: Delly, VariationHunter, BreakDancer, CNVnator, GenomeStrip, Pindel, and SSF. CNVs were merged by taking into account the confidence intervals around estimated boundaries. The merged set was genotyped across the entire population by GenomeStrip and filtered to remove redundancy and low quality sites based on genotype information. Genotypes were then updated though the integrated imputation of all variants (CNVs, SNPs, indels, etc.) with the MNV tool. The resulting genotypes were estimated to have a very low 3.1% false positive rate. Array-based experimental validation of CNV calls and PCR-based validation of CNV breakpoints were carried out by different contributors to the 1000 Genomes Project. CNVs genotyped as existing in NA12878 and not within 1000 Genomes Project described regions of VDJ recombination were selected to comprise our NA12878 gold standard CNV call set [Additional file 5: Spreadsheet 1].

A gold standard set of CNVs was similarly generated for the genome of NA10851, the aCGH control genome used in this study. 876 CNVs are common to the genomes of both NA12878 and NA10851 and are therefore are not expected to be detectable by aCGH platforms in this study.

Additionally, we generated an unfiltered CNV call set for NA12878 using1000 Genome Project deep sequencing data (2×250 bp paired-end sequencing to 60× coverage on Illumina HiSeq available at ftp://ftp-trace.ncbi.nih.gov/1000genomes/ftp/phase3/data/NA12878/high_coverage_alignment) and read-depth analysis by the CNVnator algorithm [14]. This set contained 4293 total CNV calls and 4047 autosomal CNV calls [Additional file 5: Spreadsheet 1]. Of these CNVnator calls, 609 total and 587 autosomal calls overlapped a gold standard call by 50% reciprocally. A further 137 autosomal CNVnator calls overlapped a gold standard call by less than 50% reciprocally.

### Generation of NA12878 CNV call sets

For each array design raw data from two technical replicate experiments using NA12878 DNA were obtained directly from the manufacturer or a manufacturer-recommended service provider and analyzed independently. Two CNV call sets were generated for each array design; one based on the manufacturer recommended platform specific software and another based on the platform agnostic software Nexus Copy Number 7.5 (BioDiscovery, Hawthorne CA 90250, U.S.A.) [Additional file 5: Spreadsheet 1]. The details of each analysis are described below. All chromosomal coordinates for the resulting CNV calls are based on hg19.

#### Affymetrix arrays

Raw data from hybridizations carried out on the Affymetrix SNP 6.0 and CytoScan HD arrays was obtained in the form of.cel files from published data (first replicate of SNP 6.0), from Affymetrix public data (second replicate of SNP6.0), and from Affymetrix (Santa Clara, CA 95051, U.S.A.) using NA12878 DNA. The platform specific software CNV call set for the first replicate of the SNP 6.0 array was obtained from published data [19]. The second replicate of the SNP 6.0 array was only analyzed using Nexus software as per the Affymetrix recommendation for analysis of this array. The platform specific software CNV call sets for the CytoScan HD array were obtained using the ChAS software (Affymetrix Inc. Santa Clara, CA 95051, U.S.A.) with default settings against a reference composed of a female and male HapMap sample (NA10847 and NA10851 respectively).

The Nexus call sets were generated using the SNP-FASST2 Segmentation Algorithm. The significance threshold for segmentation was set at 10^−9^ also requiring a minimum of three probes per segment and a maximum probe spacing of 1000 kb between adjacent probes before breaking a segment. The log ratio thresholds for single copy gain and single copy loss were set at 0.2 and −0.2, respectively. The log ratio thresholds for two or more copy gain and homozygous loss were set at 0.7 and −1.1 respectively. The Homozygous Frequency Threshold was set to 0.9. The Homozygous Value Threshold was set to 0.8. The Heterozygous Imbalance Threshold was set to 0.4. The minimum LOH length was set at 1 kb and minimum SNP probe density, at 0 probes/Mb. The final CNV call sets were generated by filtering the total set of predicted genomic aberrations for each array in the Nexus software and exporting the CNVs. This was done because Nexus reports LOH and Allelic Imbalance separately from losses and gains. Thus, LOH regions that are also copy number loss, allelic imbalance regions that are also copy number events, LOH regions that are not covered by copy number events, and allelic imbalance regions that are not covered by copy number events were removed.

#### Agilent arrays

Raw data from aCGH experiments carried out on the Agilent 1×1M-CGH, 1×1M-High Resolution, 2×400K-CNV, 2×400K-CGH, and 4×180K-CGH arrays using NA12878 as the test DNA and NA10851 as the control DNA were obtained from service providers in the form of feature extraction files.

The platform specific software CNV call sets were obtained by generating Interval Based Reports in the Agilent Genomic Workbench 7.0.4.0 software package (Agilent Technologies, Santa Clara CA 95051, U.S.A.) using default settings.

The Nexus call sets were generated using the FASST2 Segmentation Algorithm. The significance threshold for segmentation was set at 10^−7^, 10^−6^, 10^-5^ for the 1×1M, 2×400K and 4×180K arrays respectively, also requiring a minimum of 3 probes per segment and a maximum probe spacing of 1000 kb between adjacent probes before breaking a segment. The log ratio thresholds for single copy gain and single copy loss were set at 0.2 and −0.2, respectively. The log ratio thresholds for two or more copy gain and homozygous loss were set at 0.8 and −1.1 respectively. The final call sets were exported as text files from the software.

#### Illumina arrays

Raw data from experiments carried out on the HumanOmni5Exome v1, HumanOmni5-4v1, HumanOmni25Exome 1, HumanOmni25-8v1-1, HumanOmniExpressExome 1.2, HumanOmniExpress-24v1-0, HumanCoreExome v1.1, CytoSNP-850 k, and PsychArray using NA12878 DNA were obtained from Illumina Inc. (San Diego CA 92122, U.S.A.) in the form of.idat files.

The platform specific software CNV calls were obtained using the CNVpartition 3.2.0 algorithm with default settings in the Genome Studio 2011.1 software package (Illumina, San Diego CA 92122, U.S.A.). The Illumina-supplied analysis files used in Genome Studio are specified in Additional file 6: Table S3 for each array. Genomic regions flagged as having loss of heterozygosity and copy number of two, were removed from the final list of CNV calls from CNVpartition output.

Final reports were also generated in Genome Studio for use in Nexus CNV calling. The Nexus call sets were generated using the SNP-FASST2 Segmentation Algorithm. The significance threshold for segmentation was set at 10^−10^ for the Omni5, 10^−9^ for the Omni5Exome, Omni2.5Exome, and Omni2.5, 10^−8^ for the Omni1, and 10^−6^ for all other arrays, also requiring a minimum of 3 probes per segment and a maximum probe spacing of 1000 kb between adjacent probes before breaking a segment. The log ratio thresholds for single copy gain and single copy loss were set at 0.2 and −0.2, respectively. The log ratio thresholds for two or more copy gain and homozygous loss were set at 0.7 and −1.1 respectively. The Homozygous Frequency Threshold was set to 0.9. The Homozygous Value Threshold was set to 0.8. The Heterozygous Imbalance Threshold was set to 0.4. The minimum LOH length was set at 1 kb and the minimum SNP probe density at 0 probes/Mb. The final CNV call sets were produced by invoking two separate filtering schemes on the total set of predicted copy number aberrations for each array in the Nexus software, exporting the CNVs of each individual filtering scheme, and concatenating the two resulting CNV lists. This was done to ensure that only CNVs that were supported by both log ratio and B allele frequency evidence were included. In the first filtering scheme the following were removed: LOH regions that are also copy number loss, losses not covered by an allelic event, allelic imbalance regions that are also copy number events, LOH regions that are not covered by copy number events, allelic imbalance regions that are not covered by copy number events, and gains not covered by an allelic event. In the second filtering scheme the following were removed: LOH regions that are also copy number loss, allelic imbalance regions that are also copy number events, one copy loss, LOH regions that are not covered by copy number events, allelic imbalance regions that are not covered by copy number events, and one copy gains.

### Analysis of Illumina Infinium Multi-Ethnic Global-8 v1.0 array

Using our standard procedure, i.e. analysis with both the Nexus and CNVPartition algorithms, on data obtained from the Illumina Infinium Multi-Ethnic Global-8 v1.0 array (MEGA-EX), no CNVs were called in the genome of NA12878. This 1.7 million SNP array is designed to provide insights from diverse populations by powerfully detecting common and rare variants across the 5 most commonly studied subpopulations including African, Admixed American, East Asian, European, and South Asian. Using the QuantiSNP algorithm, 11 and 16 high confidence CNVs (Max Log BF > 10) were called in two replicates. Of these 4 and 5 CNVs respectively met the 50% reciprocal overlapping criteria with a gold standard CNV. This limited performance of the Multi-Ethnic Global array for CNV calling on the genome of the model European sample, NA12878, may be due to the number and distribution of contiguously informative SNPs on this array for European samples. We found that 12% of all SNPs on this array were found to be heterozygous in NA12878 compared to 19% and 21% of the SNPs on the similarly-sized Illumina HumanOmni2.5 and HumanOmni1Quad arrays. In any case, we did not pursue a deeper analysis of the performance of the Multi-Ethnic Global array for CNV calling in in this study. It is possible that alternative software solutions could be found to achieve more increased CNV detection with this array.

## Additional files

Additional file 1: Table S1. Overlap of CNV calls from two different algorithms for each array platform with gold standard and CNVnator CNVs. Summarizes the number of overlapping CNV calls from two different algorithms for each array platform with gold standard and CNVnator CNVs using the various overlap criteria. (XLSX 37 kb)

Additional file 2: Figure S1. Effects of tuning parameters in Nexus software on overlap of autosomal CNV calls with gold standard CNVs for a select set of arrays. Overlap data is shown for CNV call sets of two technical replicates for the Affymetrix CytoScan HD, Agilent 2×400 K and 4×180 K, and Illumina HumanOmni1Quad, HumanOmni2.5, HumanOmni2.5Exome and HumanOmni5Exome arrays using at least two different parameter settings. The number of array CNV calls that overlap a gold standard CNV by 50% reciprocally in size is shown in blue. The number of array CNV calls that overlap a gold standard CNV by less than 50% reciprocally in size is shown in pink. The number of array CNV calls that do not overlap a gold standard CNV is shown in purple. Array calls that do not overlap a gold standard CNV at all were further analyzed as either having or not having sequencing-based confirmation using CNVnator-generated CNV calls based on the 1000 Genomes Project sequencing data for NA12878. The number of CNV calls not overlapping a gold standard CNV but with CNVnator support is shown as purple bars. The number of CNV calls not overlapping a gold standard CNV and with no CNVnator support is shown as hashed purple bars. The parameters of the Nexus algorithm are relaxed from left to right for each array. The significance threshold (SigThresh) and log R ratio (LRR) settings are specified under each pair of bars. (TIFF 26369 kb)

Additional file 3: Figure S2. Cumulative frequencies of sizes of validated and non-validated CNV calls using platform specific algorithm. Cumulative frequencies of the sizes of validated CNVs are shown in red. Cumulative frequencies of sizes of non-validated CNVs are shown in green. CNV size is plotted on a log scale. Plots are shown for all arrays with more than 50 validated CNVs called using the platform specific algorithm. *P*-values were computed using a Mann–Whitney *U* test that corrects for ties and uses a continuity correction. The *p*-values correspond to a one-sided hypothesis. (PDF 88 kb)

Additional file 4: Table S2. Array detection of gold standard deletions > 100 kb in size. Summarizes the detection of the seven gold standard CNVs > 100 kb by the different arrays including the number of arrays that detected each large CNV, which arrays detected each CNV, and possible reasons why a particular large CNV may not be detectable by certain arrays. (DOCX 114 kb)

Additional file 5: Spreadsheet 1. Gold standard and array CNV chromosomal coordinates. Spreadsheet 1 lists the chromosomal coordinates of the gold standard CNVs and the CNVs called by the arrays. These data were used to compare the CNVs called by the arrays with those in the gold standard using the overlap criteria described in the methods. (XLSX 372 kb)

Additional file 6: Table 3. Manifest and cluster files used in Genome Studio analysis of Illumina arrays. Lists the Illumina-supplied manifest and cluster files for each array that were used in Genome Studio analysis. These files were downloaded from http://support.illumina.com/array/downloads.html. (DOCX 57 kb)

## Abbreviations

aCGH: Array Comparative Genome Hybridization
CNV: Copy Number Variant
ddPCR: Digital droplet Polymerase Chain Reaction
FISH: Fluorescent In Situ Hybridization
LOH: Loss of Heterozygosity
