# Supplementary figures and images for: Comprehensive performance comparison of high-resolution array platforms for genome-wide Copy Number Variation (CNV) analysis in humans
Source: BMC Genomics. 2017 Apr 24;18:321. doi: 10.1186/s12864-017-3658-x (PMC5402652; doi:10.1186/s12864-017-3658-x)

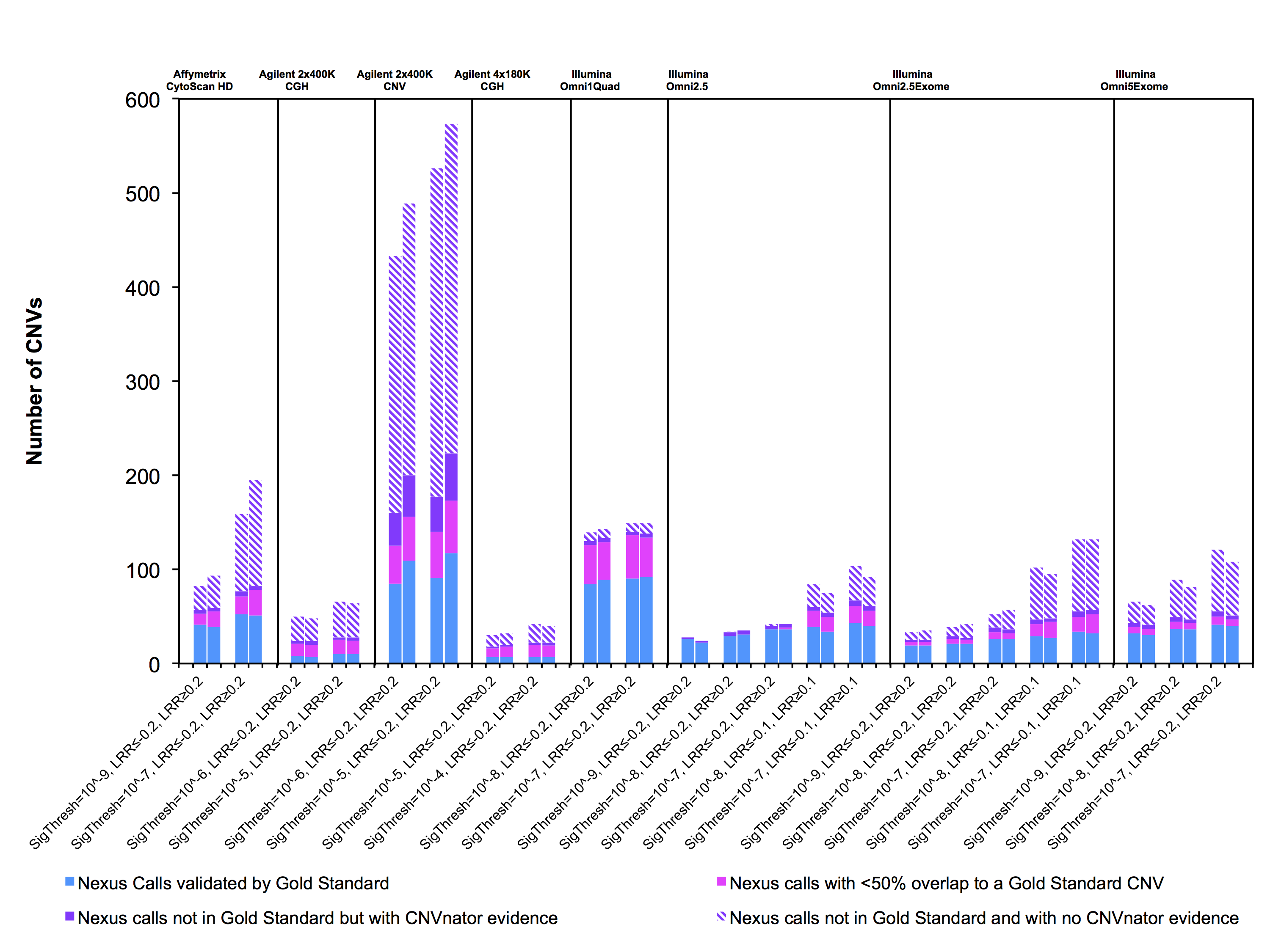

Supplement: Supplementary file 2 — Effects of tuning parameters in Nexus software on overlap of autosomal CNV calls with gold standard CNVs for a select set of arrays. Overlap data is shown for CNV call sets of two technical replicates for the Affymetrix CytoScan HD, Agilent 2×400 K and 4×180 K, and Illumina HumanOmni1Quad, HumanOmni2.5, HumanOmni2.5Exome and HumanOmni5Exome arrays using at least two different parameter settings. The number of array CNV calls that overlap a gold standard CNV by 50% reciprocally in size is shown in blue. The number of array CNV calls that overlap a gold standard CNV by less than 50% reciprocally in size is shown in pink. The number of array CNV calls that do not overlap a gold standard CNV is shown in purple. Array calls that do not overlap a gold standard CNV at all were further analyzed as either having or not having sequencing-based confirmation using CNVnator-generated CNV calls based on the 1000 Genomes Project sequencing data for NA12878. The number of CNV calls not overlapping a gold standard CNV but with CNVnator support is shown as purple bars. The number of CNV calls not overlapping a gold standard CNV and with no CNVnator support is shown as hashed purple bars. The parameters of the Nexus algorithm are relaxed from left to right for each array. The significance threshold (SigThresh) and log R ratio (LRR) settings are specified under each pair of bars. (TIFF 26369 kb) [file 12864_2017_3658_MOESM2_ESM.tiff]

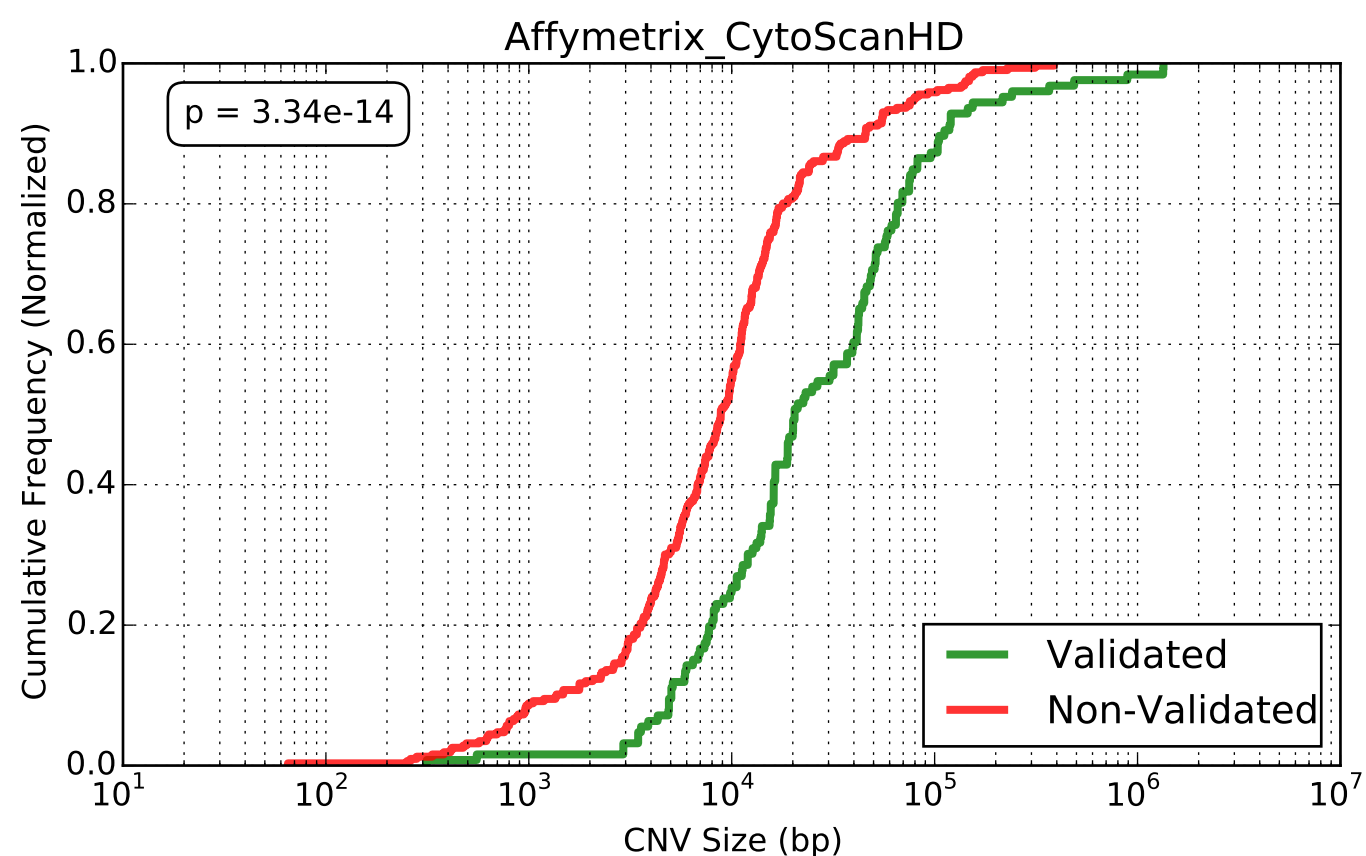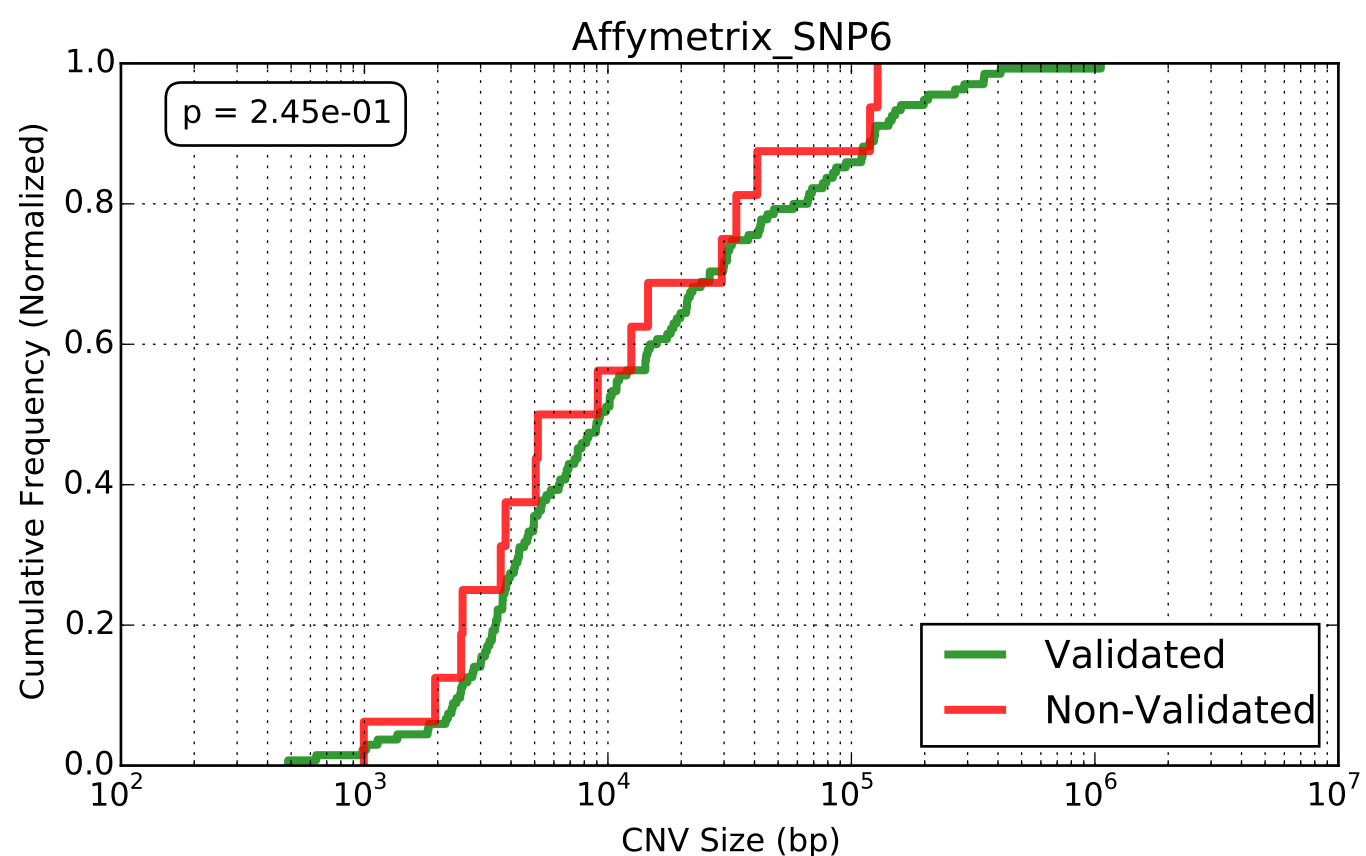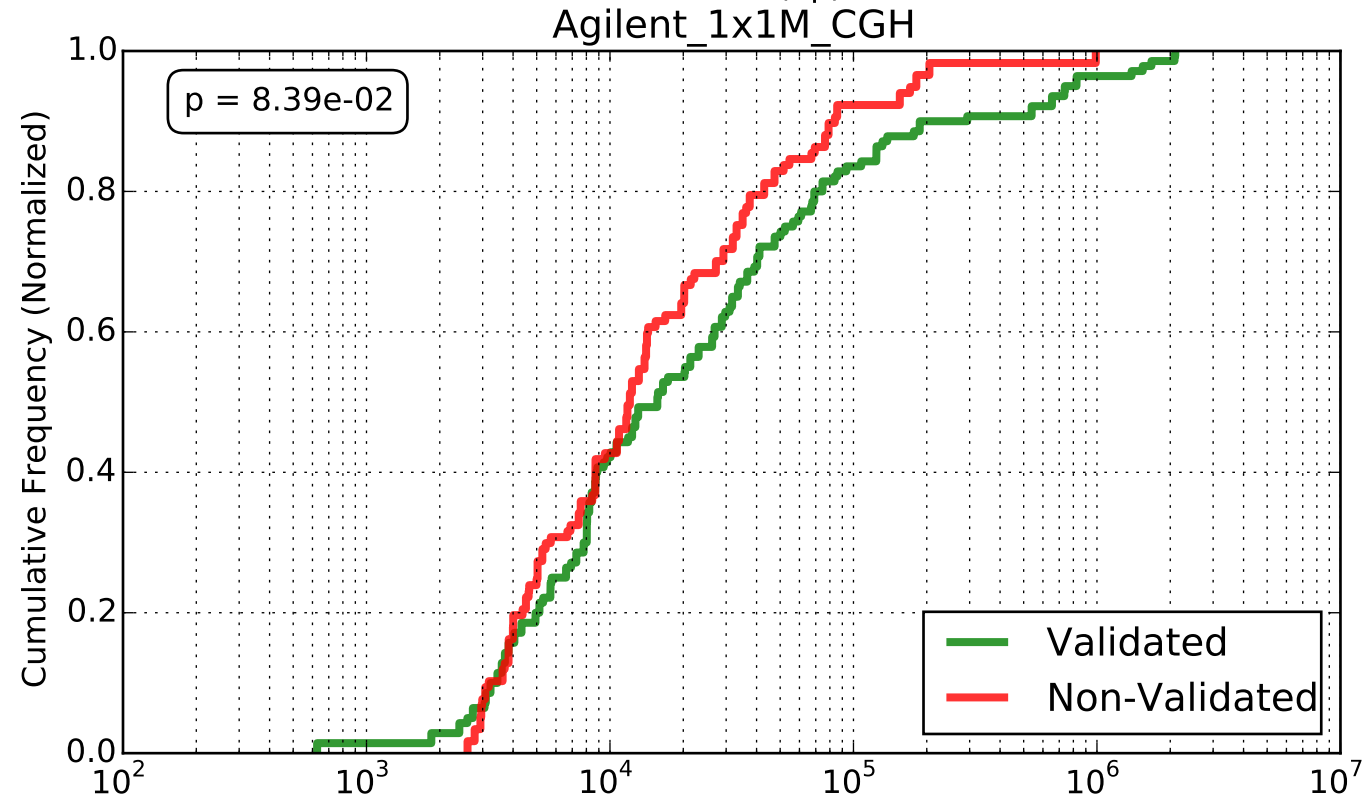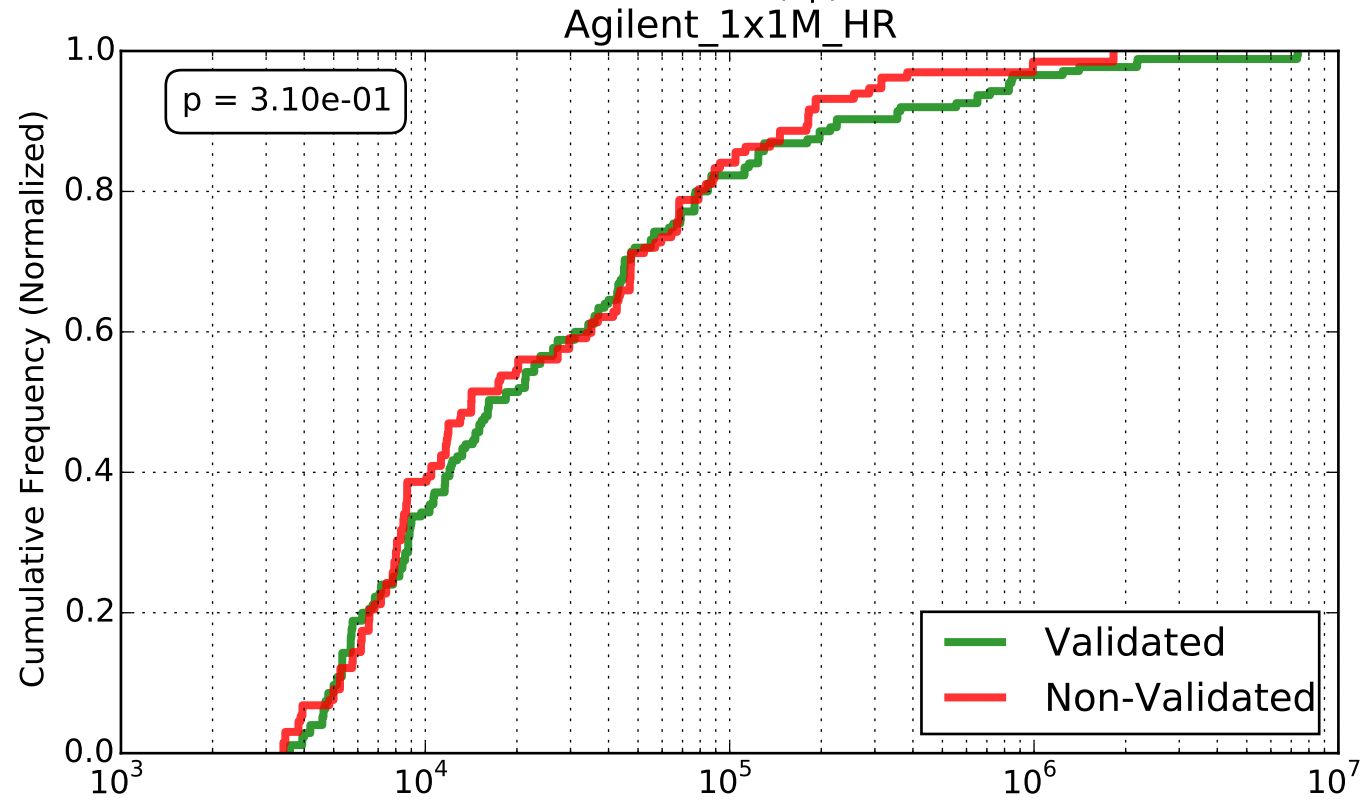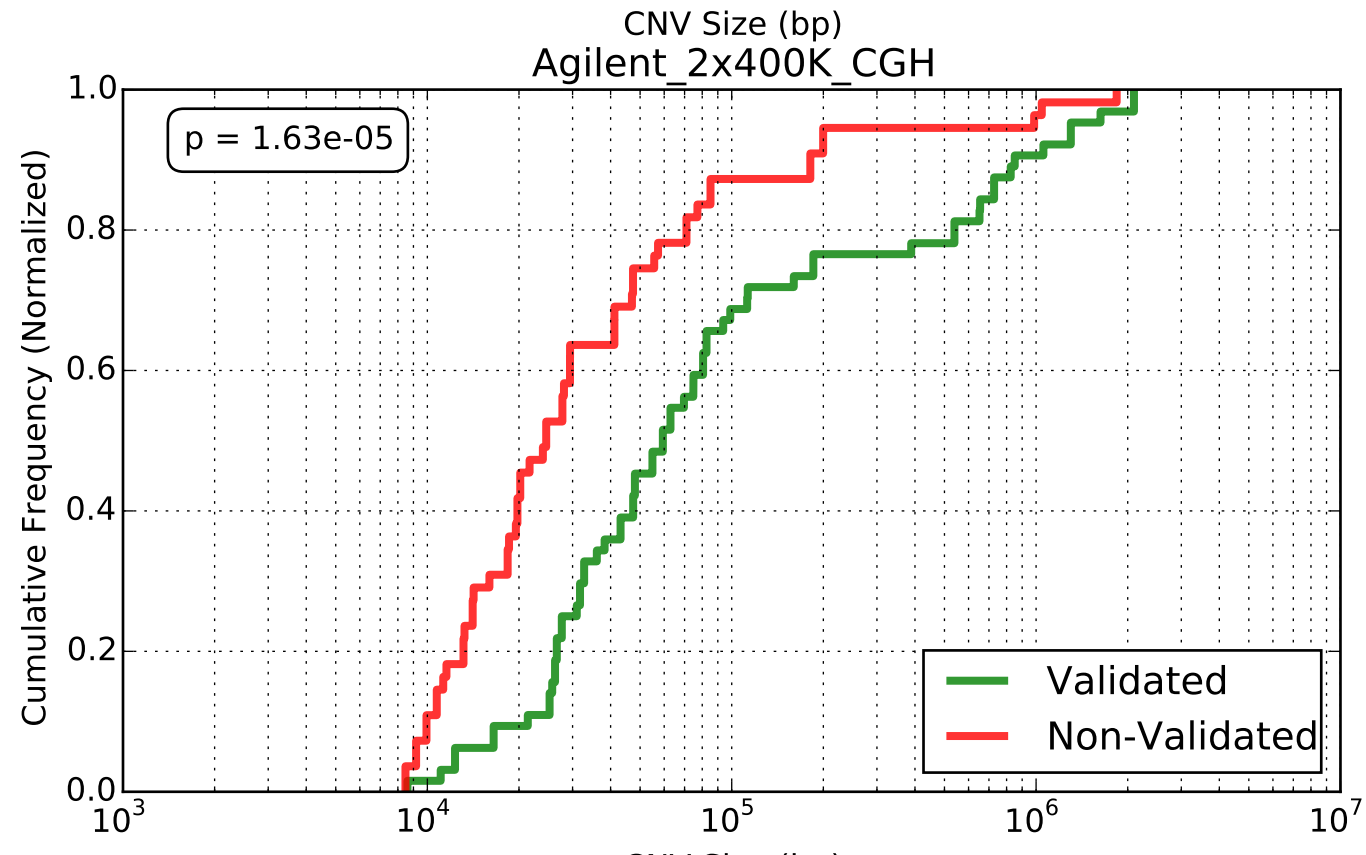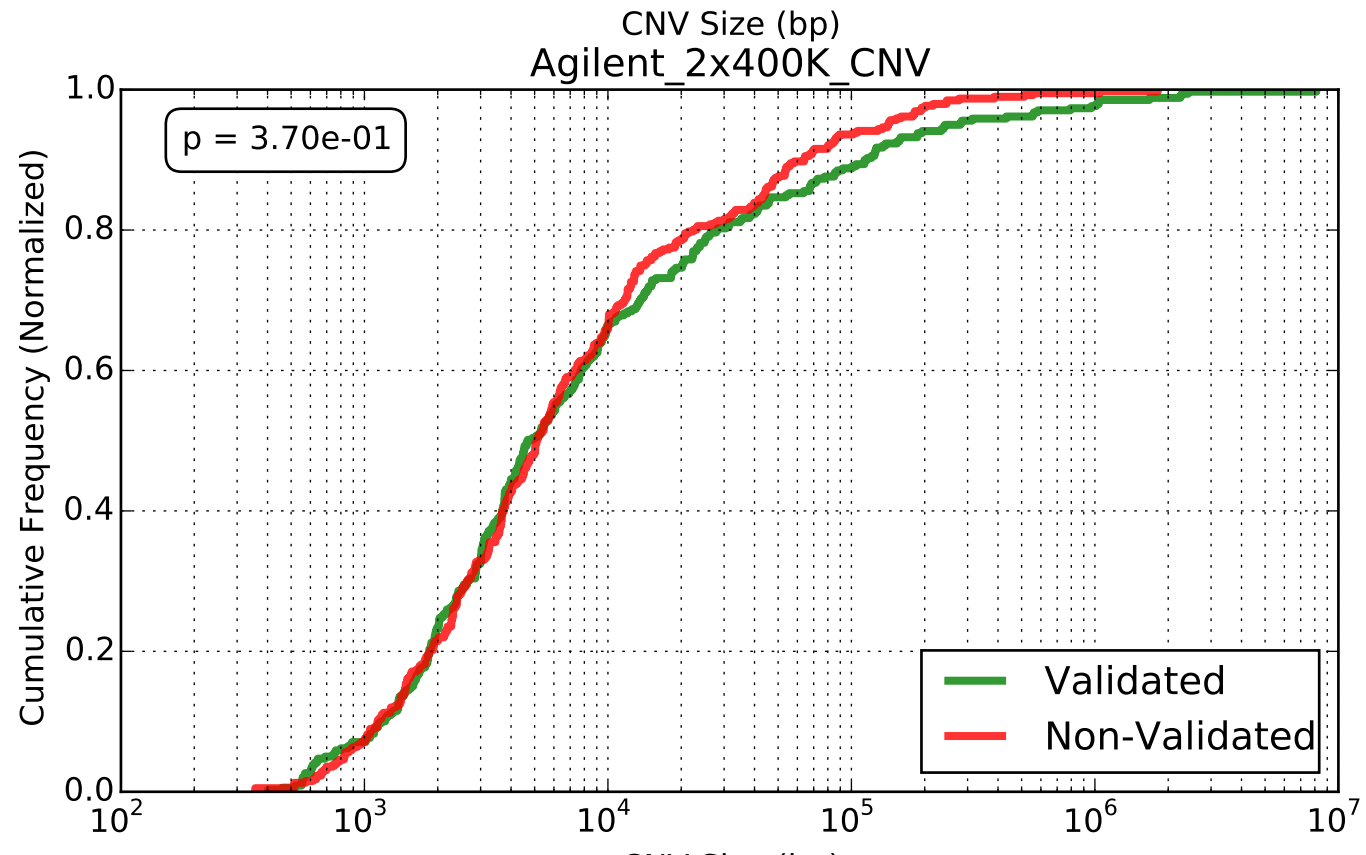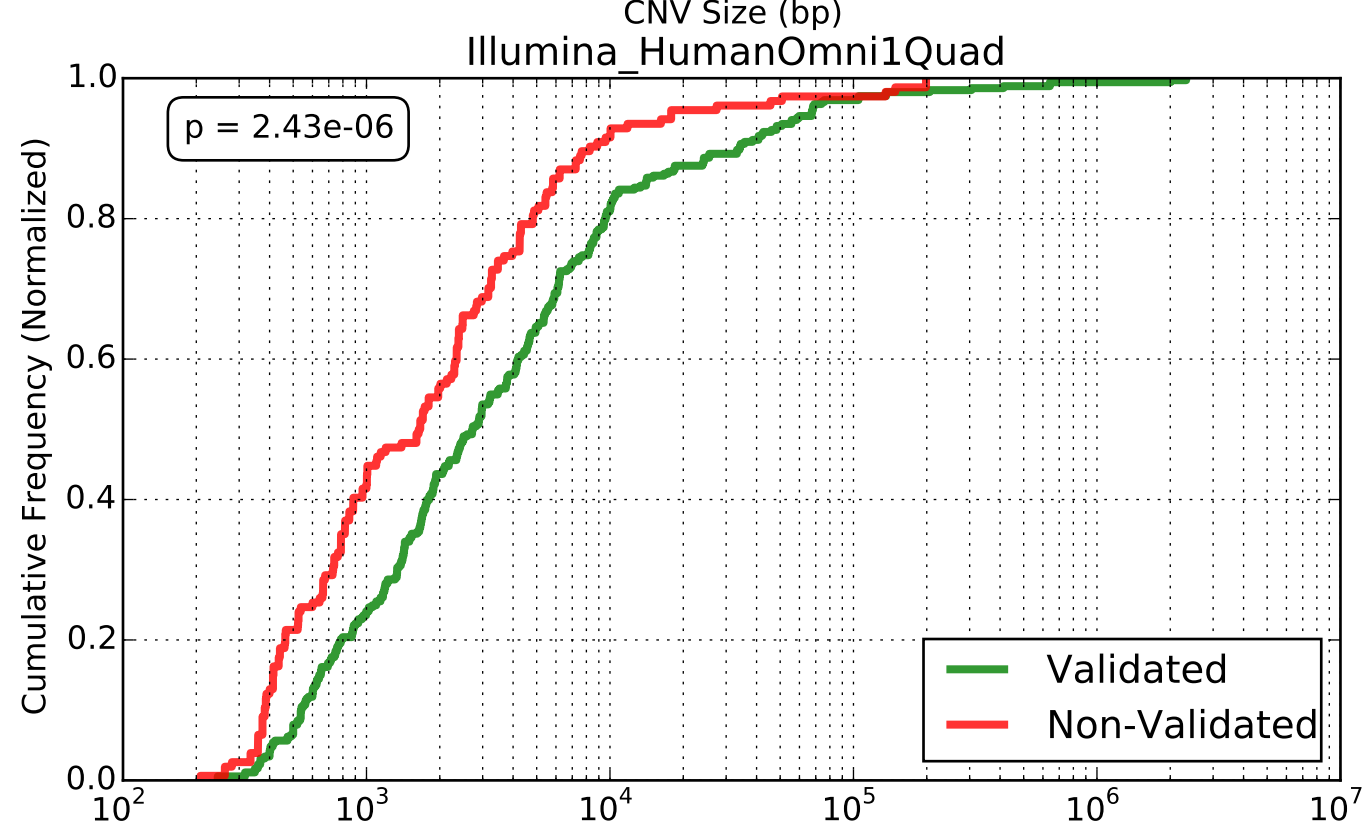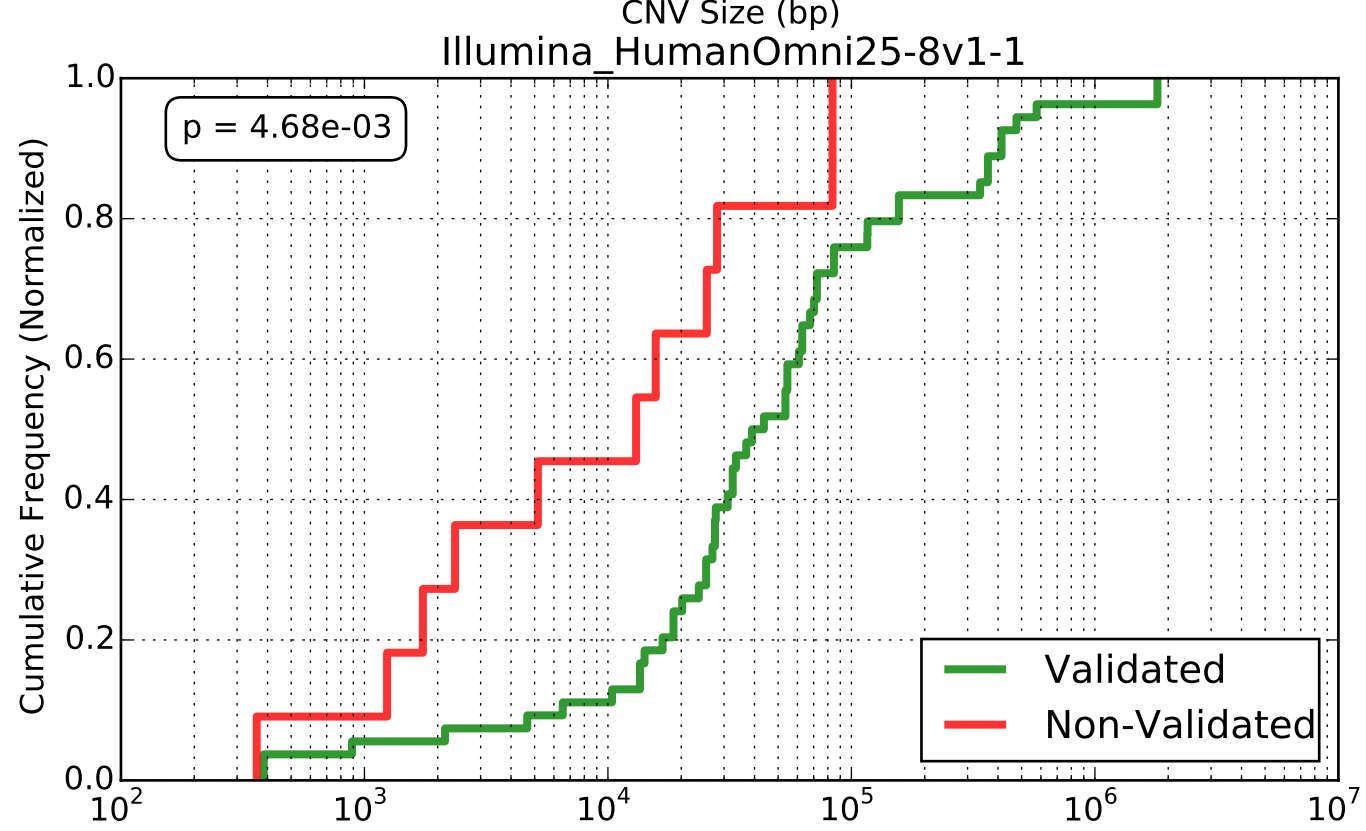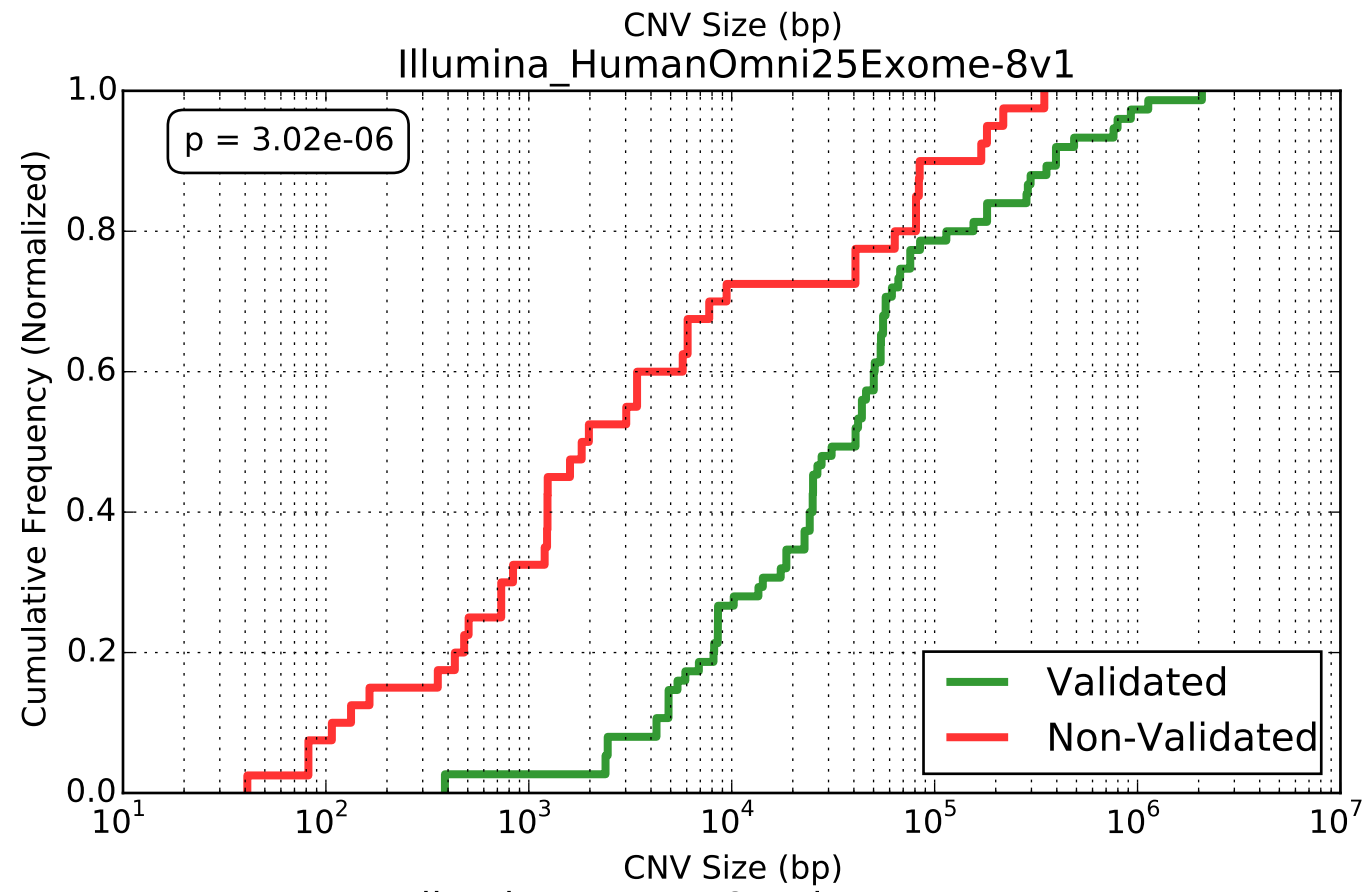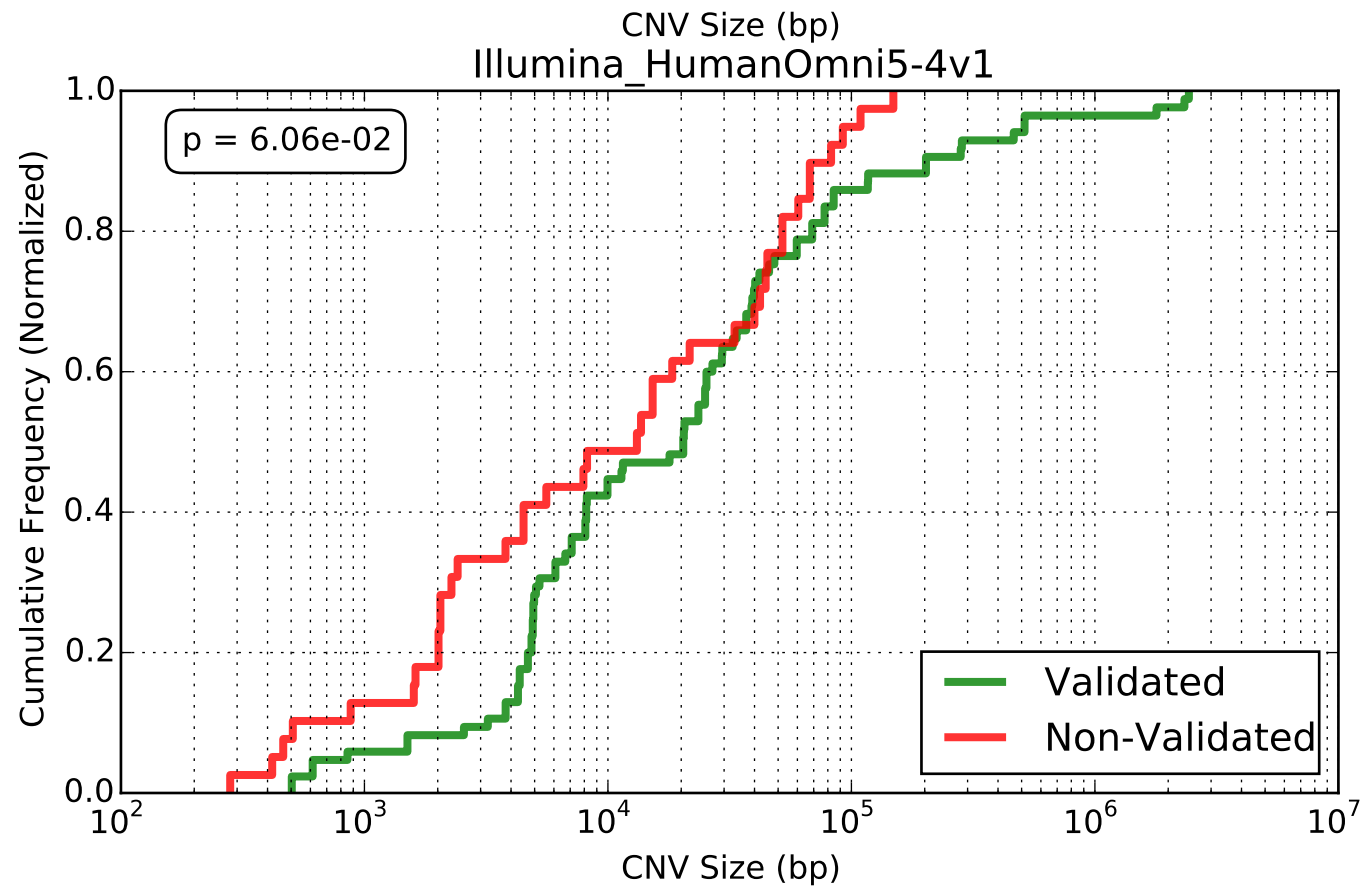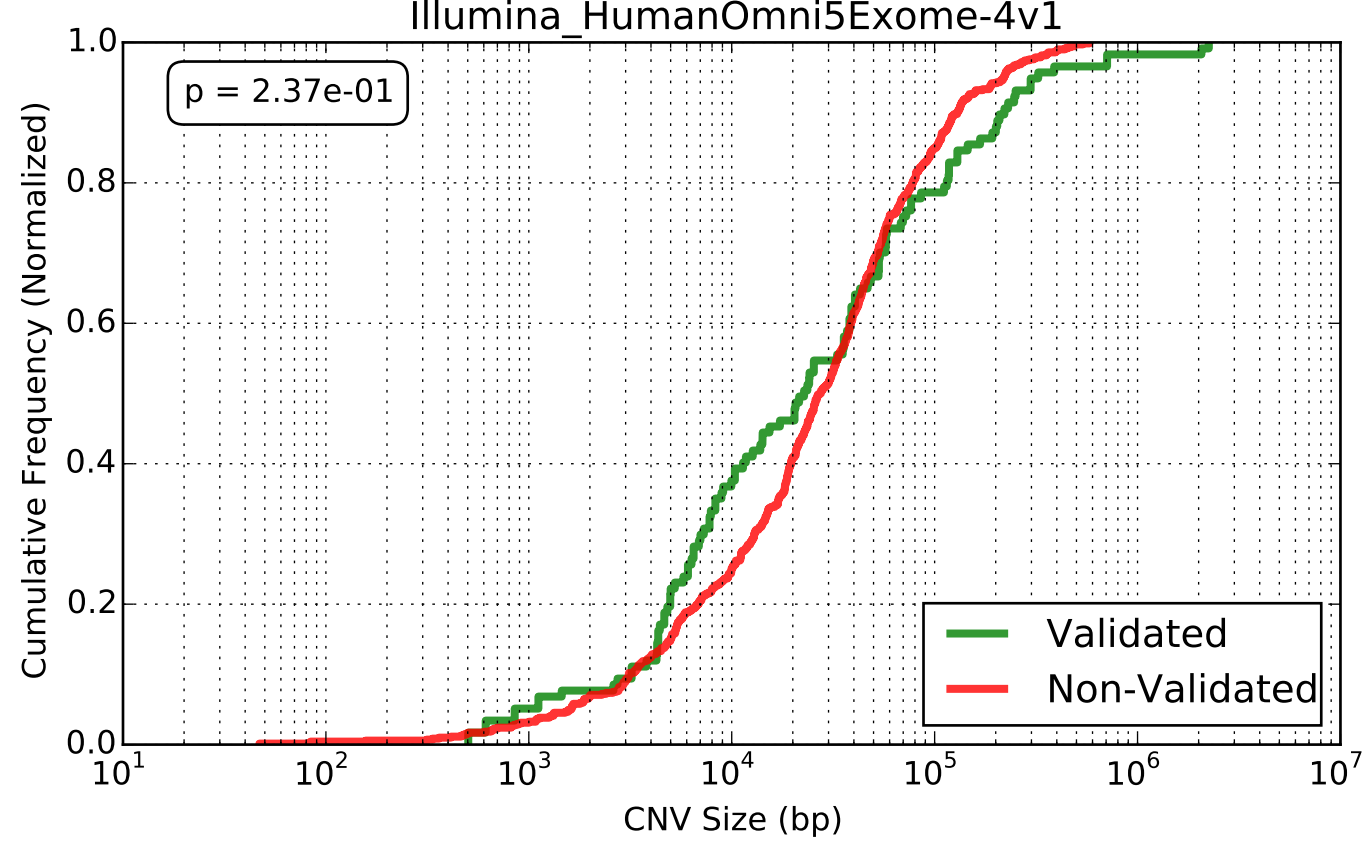

Supplement: Supplementary file 3 — Cumulative frequencies of sizes of validated and non-validated CNV calls using platform specific algorithm. Cumulative frequencies of the sizes of validated CNVs are shown in red. Cumulative frequencies of sizes of non-validated CNVs are shown in green. CNV size is plotted on a log scale. Plots are shown for all arrays with more than 50 validated CNVs called using the platform specific algorithm. P-values were computed using a Mann–Whitney U test that corrects for ties and uses a continuity correction. The p-values correspond to a one-sided hypothesis. (PDF 88 kb) [file 12864_2017_3658_MOESM3_ESM.pdf]
