# Supplementary material for: Comprehensive performance comparison of high-resolution array platforms for genome-wide Copy Number Variation (CNV) analysis in humans
Source: BMC Genomics. 2017 Apr 24;18:321. doi: 10.1186/s12864-017-3658-x (PMC5402652; doi:10.1186/s12864-017-3658-x)
Supplement: Supplementary file 4 — Array detection of gold standard deletions > 100 kb in size. Summarizes the detection of the seven gold standard CNVs > 100 kb by the different arrays including the number of arrays that detected each large CNV, which arrays detected each CNV, and possible reasons why a particular large CNV may not be detectable by certain arrays. (DOCX 114 kb) [file 12864_2017_3658_MOESM4_ESM.docx]

**Supplementary Table 2: Array detection of gold standard deletions > 100 kb in size**

| **Gold Standard Deletion**  **(Size of CNV)** | **Number of arrays detecting deletion*** | **Additional Comments** |
| --- | --- | --- |
| chr3:162514471-162625647 (111,176 bp) | 5 | Not called by any SNP/combination array, but called convincingly by all the aCGH arrays including the Agilent 180 K array. All Agilent arrays call it as duplication. This follows from the 1000 Genomes Project data, which indicates that the event is a heterozygous deletion in NA12878 and a homozygous deletion in the control genome NA10851, compared to the reference genome. There are no probes at all for any of the Illumina arrays in this region. The deletion does not lie in a segmental duplication and is not near the centromere or telomeres. |
| chr4:69375591-69491543 (115,952 bp) | 7 | Found by Affymetrix and Illumina HumanOmni2.5Quad and larger arrays. The remaining arrays do not have enough probes in the region. The Agilent 400K CNV has enough probes but only one replicate calls the CNV as a high copy duplication. According to the 1000 Genomes Project data, both NA12878 and NA10851 have homozygous deletions at this locus compared to the reference genome. |
| chr4:70122981-70231746 (108,765 bp) | 5 | Found by Affymetrix SNP6.0 array and Illumina HumanOmni2.5Exome and larger. The Omni2.5Quad does not call this even though it has the same probes plus two additional probes as the Omni2.5Exome array in this region. The remaining arrays do not have enough probes in the region. The Agilent 400K CNV has enough probes but only one replicate calls the CNV as a high copy duplication. According to the 1000 Genomes Project data, both NA12878 and NA10851 have heterozygous deletions at this locus compared to the reference genome. |
| chr6:78892808-79053430 (160,622 bp) | 9 | Found by Affymetrix arrays and Illumina OmniExpressExome and above. Not found by any Agilent array except one replicate of the Agilent 2x400K-CNV array, which calls the CNV as a high copy duplication. This follows from the 1000 Genomes Project data, which indicates that both NA12878 and NA10851 have the same genotype at this locus, a heterozygous deletion compared to the reference genome. This CNV is polymorphic in the 1000 Genomes Project populations. |
| chr8:39195825-39389230 (193,405 bp) | 5 | Found by the Affymetrix arrays and Illumina Omni2.5 and Omni1Quad arrays. The Illumina Omni2.5Exome and higher have enough probes in the region but do not call the CNV. Not found by any Agilent array even though most arrays have a decent number of probes in the region except one replicate of the Agilent 2x400K-CNV array, which calls the CNV as a high copy duplication. This follows from the 1000 Genomes Project data, which indicates that both NA12878 and NA10851 have the same genotype at this locus, a heterozygous deletion compared to the reference genome. |
| chr11:4248265-4353229 (104,964 bp) | 3 | Found by the Affymetrix SNP 6.0, Agilent 2x400K-CNV, and one replicate of the 1x1M HR. All other arrays have very few or no probes in this region. |
| chr19:20595835-20717950 (122,115 bp) | 16 | Called by all arrays except the Illumina Psych array, which does not have enough probes in that region. |

***A deletion is considered detected if it was called by at least one replicate using Nexus.**
