# Supplementary material for: Comprehensive performance comparison of high-resolution array platforms for genome-wide Copy Number Variation (CNV) analysis in humans
Source: BMC Genomics. 2017 Apr 24;18:321. doi: 10.1186/s12864-017-3658-x (PMC5402652; doi:10.1186/s12864-017-3658-x)
Supplement: Supplementary file 6 — Manifest and cluster files used in Genome Studio analysis of Illumina arrays. Lists the Illumina-supplied manifest and cluster files for each array that were used in Genome Studio analysis. These files were downloaded from http://support.illumina.com/array/downloads.html. (DOCX 57 kb) [file 12864_2017_3658_MOESM6_ESM.docx]

**Supplementary Table 3: Manifest and cluster files used in Genome Studio analysis of Illumina arrays.**

| **Array** | **Manifest file (.bpm)** | **Cluster file (.egt)** |
| --- | --- | --- |
| HumanOmni5Exome v1 | humanomni5exome-4v1_a.bpm | HumanOmni5MExome-4v1_Gentrain-262_reClustered.egt |
| HumanOmni5-4v1 | humanomni5-4v1_c.bpm | humanomni5m-4v1_b.egt |
| HumanOmni25Exome 1 | humanomni25exome-8v1_a.bpm | humanomni25exome-8v1_a.egt |
| HumanOmni25-8v1-1 | humanomni25m-8v1-1_b.bpm | humanomni2-5m-8v1-1_b.egt |
| HumanOmniExpressExome 1.2 | humanomniexpressexome-8v1-2_a.bpm | HumanOmniExpressExome-8v1-2_A.egt |
| HumanOmniExpress-24v1-0 | humanomniexpress-24v1-0_a.bpm | humanomniexpress_24v1-0_a.egt |
| HumanCoreExome v1.1 | HumanCoreExome-12v1-1_B.bpm | humancoreexome-12v1-1_a.egt |
| CytoSNP-850K | cytosnp-850k_b.bpm | CytoSNP-850K_B.egt |
| PsychArray | PsychArray_A.bpm | PsychArray_A_ClusterFile.egt |

Manifest and cluster files were downloaded from http://support.illumina.com/array/downloads.html
